# Acetyl-4′-phosphopantetheine is stable in serum and prevents phenotypes induced by pantothenate kinase deficiency

**DOI:** 10.1038/s41598-017-11564-8

**Published:** 2017-09-12

**Authors:** Ivano Di Meo, Cristina Colombelli, Balaji Srinivasan, Marianne de Villiers, Jeffrey Hamada, Suh Y. Jeong, Rachel Fox, Randall L. Woltjer, Pieter G. Tepper, Liza L. Lahaye, Emanuela Rizzetto, Clara H. Harrs, Theo de Boer, Marianne van der Zwaag, Branko Jenko, Alen Čusak, Jerca Pahor, Gregor Kosec, Nicola A. Grzeschik, Susan J. Hayflick, Valeria Tiranti, Ody C. M. Sibon

**Affiliations:** 1 Division of Molecular Neurogenetics, IRCCS Foundation Neurological Institute “C.Besta” Via Temolo 4, 20126 Milano, Italy; 2 Department of Cell Biology, University Medical Center Groningen, University of Groningen, Ant. Deusinglaan 1, 9713 AV Groningen, The Netherlands; 3 Department of Biochemistry, Stellenbosch University, Stellenbosch, 7600 South Africa; 4 Departments of Molecular & Medical Genetics and Pathology, Oregon Health & Science University, Portland, OR 97239 USA; 5 Department of Chemical and Pharmaceutical Biology, University of Groningen, Ant. Deusinglaan 1, 9713 AV Groningen, The Netherlands; 6 Clinical Pathology and Medical Genetics Unit, Foundation IRCCS-Neurological Institute “Carlo Besta”, Milano, Italy; 7 Analytical Biochemical Laboratory (ABL), WA Scholtenstraat 7, 9403 AJ Assen, The Netherlands; 8 Acies Bio d.o.o., Tehnološki park 21, 1000 Ljubljana, Slovenia; 9 Laboratory of Organic and Bioorganic Chemistry, Department of Physical and Organic Chemistry, Jožef Stefan Institute, Jamova 39, 1000 Ljubljana, Slovenia

## Abstract

Coenzyme A is an essential metabolite known for its central role in over one hundred cellular metabolic reactions. In cells, Coenzyme A is synthesized *de novo* in five enzymatic steps with vitamin B5 as the starting metabolite, phosphorylated by pantothenate kinase. Mutations in the pantothenate kinase 2 gene cause a severe form of neurodegeneration for which no treatment is available. One therapeutic strategy is to generate Coenzyme A precursors downstream of the defective step in the pathway. Here we describe the synthesis, characteristics and *in vivo* rescue potential of the acetyl-Coenzyme A precursor S-acetyl-4′-phosphopantetheine as a possible treatment for neurodegeneration associated with pantothenate kinase deficiency.

## Introduction

**Figure 1:**
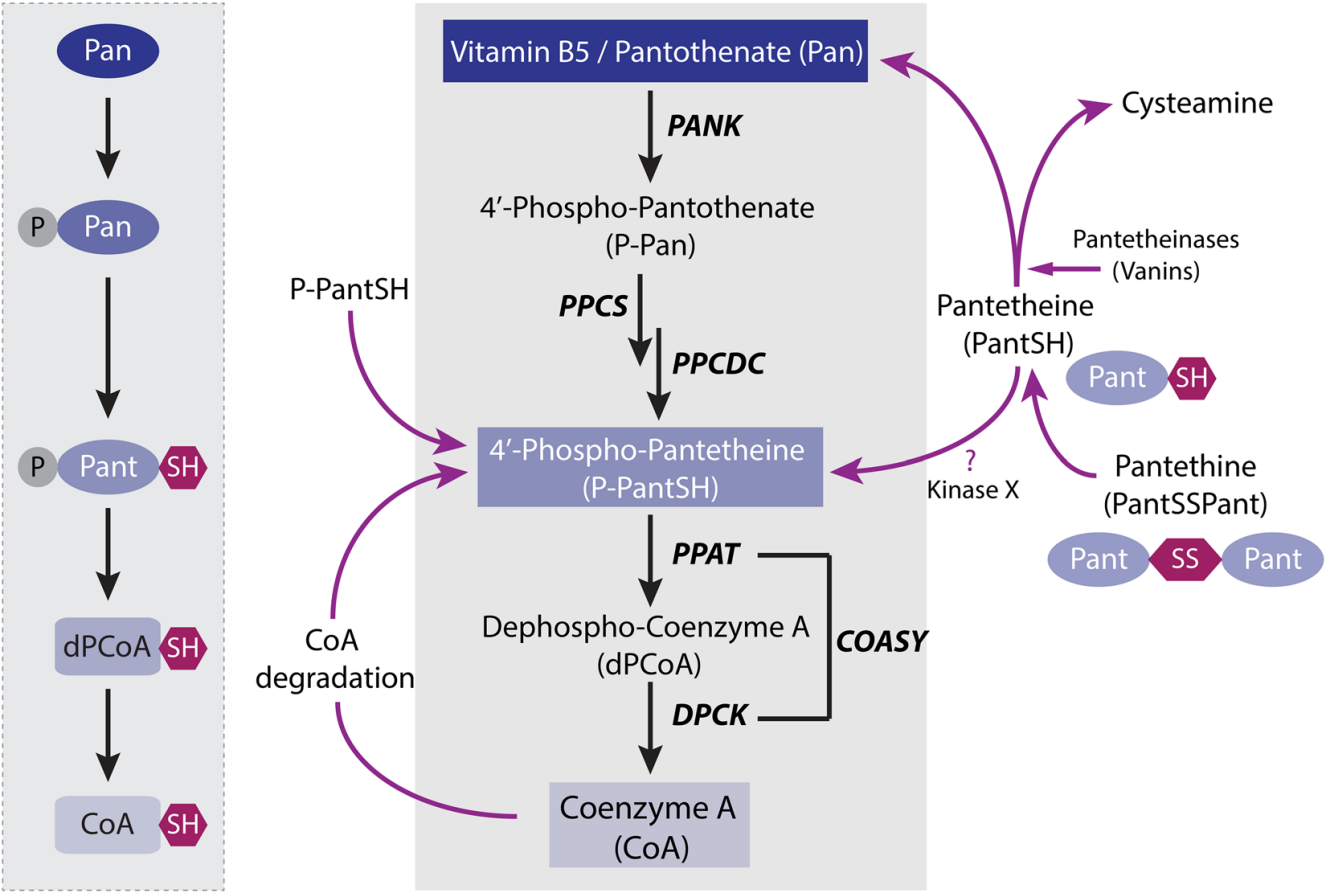
CoA metabolism. CoA *de novo* biosynthesis. Vitamin B5, or pantothenate, is taken up and intracellularly converted to CoA sequentially by the enzymes PANK, PPCS, PPCDC, COASY (PPAT and DPCK). Order and abbreviations of starting, intermediate and end products (Pan, P-Pan, P-PantSH, dPCoASH and CoASH) are provided on the left. Several salvage pathways to this route exist. Externally provided CoA can be converted into P-PantSH, which can be taken up by cells and intracellularly converted to CoA^29^. Externally provided PantSH can rescue a CoA-depleted phenotype^18, 31^ via unknown mechanisms including a possible ‘kinase X’ that phosphorylates PantSH to form P-PantSH. Pant-SS-Pant can be converted into PantSH. PantSH can be degraded into Pan and cysteamine by pantetheinases or vanins. PANK = pantothenate kinase; PPCS = phosphopantothenoylcysteine synthetase; PPCDC = phosphopanthenoylcysteine-decarboxylase; PPAT = phosphopantetheine adenylyltransferase; PPCK = dephospho-CoA kinase. Pan = pantothenate; P-Pan = 4′-phosphopantothenate; Pant-SS-Pant = pantethine; PantSH = pantetheine; P-PantSH = 4′-phosphopantetheine; dPCoASH = dephosphoCoA; CoASH = Coenzyme A.

Coenzyme A (CoA), an essential metabolic cofactor known for over 70 years, is able to carry and transfer acetyl and other acyl groups to other molecules^1^. CoA is involved in energy and fatty acid metabolism^2, 3^, and levels of CoA and acetyl-CoA influence numerous biological processes including cell growth, cell death, signal transduction, epigenetics, protein acetylation and more (for reviews see^4–6^. One pathway for cells and organisms to synthesize CoA requires the uptake of vitamin B5, alternatively known as pantothenate (Pan), which is converted by a series of enzymatic reactions into CoA (Fig. 1). The five enzymes that carry out these reactions are pantothenate kinase (PANK), 4′-phosphopantothenoylcysteine synthetase (PPCS), 4′-phosphopantethenoylcysteine decarboxylase (PPCDC), phosphopantetheine adenylyltransferase (PPAT) and dephospho-CoA kinase (DPCK)^2^. This highly conserved pathway, referred to as the canonical *de novo* CoA biosynthesis pathway (Fig. 1), is present in nearly all organisms^2, 7–12^. It should be noted that humans and mice have 4 genes (*PANK1–4*) encoding PANK enzymes and only *PANK1–3* encode proteins with functional pantothenate kinase activities (reviewed in ref. 2). In addition, the two final synthesis steps requiring PPAT and DPCK activity in humans, mice and *Drosophila melanogaster* are catalyzed by a bifunctional CoA synthase enzyme that is referred to as COASY^8, 9, 13–15^. Two hereditary diseases have been linked to inborn errors of CoA metabolism^14, 16^, further underscoring the essential role of CoA in living organisms. Patients that carry mutations in *PANK2* or *COASY* suffer from progressive movement disorders, characterized by severe dystonia and iron accumulation in the basal ganglia^17^. The disorders are referred to as Pantothenate Kinase-Associated Neurodegeneration (PKAN) and COASY Protein-Associated Neurodegeneration (CoPAN). Mutations in *PANK2* and *COASY* are associated with similar symptoms arising from damage to a common brain region. This observation bolsters the evidence of impaired CoA homeostasis in the pathophysiology of both diseases. There are currently no treatments available for either PKAN or CoPAN; however, concerted efforts are being made to develop rational therapeutics that bypass the biochemical defects.

Towards that end, a *Drosophila melanogaster* model for PKAN that recapitulates important phenotypic features of neurodegeneration has been used to develop candidate treatments. Recently it was demonstrated that the CoA precursor pantethine (PantSSPant, the disulfide form of pantetheine (PantSH)) can replenish decreased levels of CoA under conditions of impaired function of PANK enzymatic activity in a multicellular organism. Importantly, the addition of Pan did not alleviate any CoA defects in this study^18^. After this initial study it was also demonstrated that PantSSPant addition to the food of *PANK2* ^*−/−*^ knockout mice rescued various phenotypes induced by a high fat diet^19^. Consistent with the results in *Drosophila*, PantSSPant but not Pan was also protective in a zebrafish model for PKAN^20^. The exact rescue mechanism by PantSSPant in a *PANK* compromised background is not known. One explanation is that PantSSPant is converted into PantSH and because of the promiscuous activity of PANK, this enzyme can also phosphorylate PantSH to form 4′-phosphopantetheine (P-PantSH), which can be further processed by the enzymes downstream in the pathway to form CoA (Fig. 1)^3, 21^. Therefore the presence of residual activities of PANK in the PKAN models, or the redundant activities of other PANKs, or a yet un-identified pantetheine kinase could explain the observed rescue by PantSSPant in various PKAN models. One limitation for further development of PantSSPant or PantSH as a possible treatment for PKAN is the instability of PantSH in mammals due to the presence of pantetheinases (members of the Vanin family) in the gastrointestinal mucosa and in serum. These enzymes degrade pantetheine into Pan and the antioxidant cysteamine^22–26^ (Fig. 1). Several strategies have been explored to increase the serum-stability of PantSH and compounds similar in structure to PantSH (the so-called *N*-substituted pantothenamides) by modifications of the scissile amide bond present in pantothenamides or alternatively by modifications to the cysteamine moiety of PantSH^24, 27, 28^. These results demonstrate that the activity of pantetheinase is not hindered by various modifications of the cysteamine moiety. However, modifying the scissile bond does impair pantetheinase activity and renders these compounds resistant to degradation. Additionally, the reliance of PantSH based compounds on residual PANK, or alternative kinase activity, is a potential risk in translation of efficacy from preclinical models towards clinical use in humans.

Recently we demonstrated that in contrast to PantSH, the naturally occurring CoA precursor P-PantSH is stable in mammalian serum. In addition, P-PantSH is stable *in vivo* in mice, has membrane-crossing properties and is able to restore levels of CoA under conditions of impaired PANK activity. As a result, it rescues phenotypes induced by decreased levels of CoA such as sterility, developmental arrest, neurodegeneration and life span in *Drosophila* ^29^. Synthesis of P-PantSH at sufficient purity for biological evaluation is challenging due to the presence of a free thiol, which hinders purification and may also influence the oxidative stability after its synthesis. This is a significant consideration for potential therapeutics, which must be produced to clinical standards and be sufficiently stable for distribution. No information is yet known whether modifications of P-PantSH can influence the serum stability and/or rescue potential of this molecule; specifically modifications of the free thiol of P-PantSH. Here our aim was to design a synthesis for S-acetyl-4′-phosphopantetheine (P-PantSAc), which incorporates purification in the final acylated form only, and to investigate whether addition of an acetyl group to the thiol moiety of P-PantSH affects its serum stability and its rescue abilities of phenotypes induced by impaired CoA biosynthesis.

P-PantSAc is also of interest because it may serve to be an alternative source for both CoA and acetyl-CoA (AcCoA). This is supported by the fact that S-acetyl-pantetheine (PantSAc) can be converted to AcCoA by PanK, PPAT and DPCK from *Escherichia coli* ^30^. Here, our findings demonstrate that P-PantSAc is stable in serum and possesses rescue potential comparable to 4′-phosphopantetheine in both *in vitro* and *in vivo* models. Taken together, P-PantSAc is a promising candidate replacement therapy for neurodegeneration associated with impaired activity of *PANK2*.

## Materials and Methods

### Stability of metabolites in FCS

The stability of the PantSH, P-PantSH and PantSAc was determined by incubating each compound in fetal calf serum (Gibco; pre-incubated at 37 °C for 30 min). Each compound was incubated in a final concentration of 1 mM in the presence of 50% FCS. Stock concentrations of each compound were prepared in PBS, and control samples contained only PBS with no FCS. The samples were incubated at 37 °C for a further 30 min after which 8.33 mM TCEP was added. After incubation at room temperature for 15 min, the samples were transferred to a Millipore 3 K centrifugal filter unit and centrifuged at 14000 rpm for 15 min at 4 °C. The filtrate of each sample was transferred to HPLC vials and analyzed. HPLC analyses were performed on a Shimadzu LC-10AC HPLC, SCL-10A system controller, SIL SCL-10A system controller, SIL-10AC automatic sample injector and LC-10AT solvent delivery system. Compounds were detected by a Shimadzu SPD-20A UV/VIS detector at a wavelength of 205 nm. Signal output was collected digitally with Shimadzu Labsolution software and post-run analyses were performed. Chromatographic separation of the analytes was achieved with a Phenomenex C18 guard column (4 × 3mm) connected to a Phenomenex Synergi Hydro-RP analytical column (4.6 × 150mm; 3 µm particles) at 30 °C. The two mobile phases consisted of solvent A: 20 mM KH_2_PO_4_ buffer (pH 3) containing 0.1% (m/v) hexansulfonate and solvent B: acetonitrile. Flow rate was maintained at 1 mL/min with a slow gradient elution: 0–5 min 0% solvent B; 5–18 min 30% solvent B; 18–21 min 50% solvent B; 21–23 min 50% solvent B; 23–24 min 0% solvent B; and 24–30 min 0% solvent B for re-equilibration.

### Toxicity in HEK293 cells

HEK293 cells were maintained in dMEM (Invitrogen) supplemented with 10% FCS (Gibco) and antibiotics (penicillin/streptomycin, Invitrogen). For toxicity assays, cells were transferred to 6-well sterile culture plates with dMEM media (containing 10% FCS and antibiotics) and supplemented with either PantSH, P-PantSH or P-PantSAc to achieve a final cell concentration of 0.1 × 10^6^ cells in 3 ml final volume. Compounds were tested in varying concentrations (0–500 μM). Cells were incubated for 4 days at 37 °C, after which a manual cell count was performed to determine survival.

### Drosophila S2 Cell Culture, HoPan treatment, RNA Interference, 4′-phosphopantetheine/P-PantSAc treatment

*Drosophila* Schneider′s S2 cells were maintained as described previously^29^. For HoPan treatment, cells were cultured in Schneider’s medium with HoPan at a final concentration of 0.5 mM. P-PantSH or P-PantSAc was added to the cells for a final concentration of 100 µM. Two days after the start of treatment the cells were counted.

Synthesis of dPANK/*fbl-RNAi* constructs and treatment with RNA interference (dPANK/fbl dsRNA) was performed as described previously^31^. As a control S2 cells were treated with a irrelevant RNAi construct targeted against the human gene hMAZ. Briefly, the cells were incubated with dsRNA for 4 days to induce efficient knock-down and were then sub-cultured, with or without PPantSH (Acies Bio, >92%) or P-PantSAc (Acies Bio, >91%) at 100 µM each and maintained for an additional 3 days, after which they were counted.

### HoPan treatment of mammalian HEK293 cells in combination with P-PantSH and P-PantSAc treatment

HEK293 cells were maintained as described previously^29^. For HoPan treatment, cells were cultured in custom-made dMEM without vitamin B5 (Thermo Scientific) and supplemented with dialyzed FCS (Thermo Scientific). P-PantSH or P-PantSAc was added to the cells for a final concentration of 25 µM for 4 days in the presence of HoPan at a final concentration of 0.5 mM. Four days after the start of treatment the cells were counted.

### Drosophila S2 Cell Immunofluorescence Staining

Immunofluorescence analysis on *Drosophila* S2 cells was performed as described in detail previously^29^. Briefly, the cells were stained with rabbit anti-AcLys antibody (Cell Signaling Cat No: 9441, 1:500) as primary and goat anti-rabbit-Alexa488 antibody (Molecular Probes) as secondary antibody. Rhodamin-Phalloidin (20U/ml) (Invitrogen) was used to detect F-actin, and DNA was detected by DAPI (0.2 μg/ml) (Thermo Scientific). The samples were analyzed with a Leica fluorescence microscope with Leica software. Adobe Photoshop and Illustrator (Adobe Systems Incorporated, San Jose, California, USA) were used for image assembly.

### *Drosophila* Maintenance, HoPan Toxicity and CoA/P-PantSAc Rescue Experiment

*Drosophila melanogaster* stocks were maintained on standard cornmeal agar fly food (containing water, agar 17 g/L, sugar 54 g/L, yeast extract 26 g/L and nipagin 1.3 g/L) at 25 °C.

For the HoPan toxicity assay, *w1118* flies (Bloomington Stock Centre (Indiana University, USA)) (10 females + 5 males) were raised in vials with standard food with or without HoPan at a final concentration of 5 mM and CoA or P-PantSAc at a final concentration of 6 mM. The flies were allowed to lay eggs for 2 days, after which the adults were discarded. The resulting offspring were allowed to develop. The numbers of pupae were counted to evaluate HoPan toxicity and CoA or P-PantSAc rescue efficiency.

### Mice treatments

Animal studies were approved by the Ethics Committee of the Foundation IRCCS Neurological Institute C. Besta, in accordance with guidelines of the Italian Ministry of Health, Project No. BT4/2014. The use and care of animals followed ItalianLaw D.L. 116/1992 and EU directive 2010/63/EU. Six week-old C57BL/6 N male mice (Charles River) were housed with a stocking density of 2/3 per cage in a temperature-controlled (21 ± 2 °C) room with a 12 h light/dark cycle and 55 ± 10% relative humidity and access to food and water *ad libitum*. Mice were pre-conditioned with a low pantothenic acid (PA)-diet (Harlan 95248RC) for 2 weeks before the start of treatments and maintained on the same diet for the whole experimental period. HoPan (100 μg/g/day) and/or CoA (100 μg/g/day, SIGMA) and/or P-PantSAc (100 μg/g/day) were dissolved in water and administered by oral gavage daily using polypropylene 20G-feeding tubes (Instech). Mouse weight and food and water intakes were recorded daily. Euthanasia was carried out by cervical dislocation.

### Acylcarnitines measurement

Liver tissue was homogenized and tissue extracts prepared as previously described^32, 33^. Briefly, after addition of an internal standard control solution (0.02 μg/μl methyl-D3-carnitine; 0.01 μg/μl heptanoylcarnitine; 0.01 μg/μl undecanoylcarnitine), homogenates were treated with acetonitrile and centrifuged at 10000 X g for 10 minutes. Surpernatants were collected and mixed with hexanes to separate the organic phase containing neutral lipids and the aqueous phase containing acylcarnitines. This second phase was dried under nitrogen, derivatized with butanol and acetyl-chloride, and incubated at 63 °C for 20 minutes. Each sample was diluted and analysed by ESI/MS/MS (Electrospray Ionization Tandem Mass Spectrometry) using an AB Sciex Api2000 Triple Quadrupole. Free carnitine and acylcarnitine profile was determined using scanning for precursor ions at m/z = 85.1. Quantification was performed using Chemo View software. Protein concentration was determined by Lowry method.

### Tubulin acetylation

Livers were homogenized on ice with a glass-glass potter and lysed using RIPA buffer (50 mM Tris pH 8, 150 mM NaCl, 1% NP40, 0.5% Na-deoxycholate, 0.1% SDS, 5 mM EDTA pH 8) with addition of protease inhibitor cocktail (Roche). Proteins were quantified by BioRad protein assay according to manufacturer instructions. Equal amounts of proteins (20 μg) were resolved on a 12% SDS-polyacrilamide gel and electroblotted onto nitrocellulose membrane. Filters were incubated with mouse monoclonal anti-acetylated tubulin antibody (clone 6–11B-1, Sigma). Equal loading was verified using a mouse monoclonal anti-GAPDH antibody (clone 6C5, Millipore). Peroxidase-conjugated secondary antibodies (Amersham) were visualized using the ECL method with autoradiography film. Analysis of Tubulin and histone acetylation levels of Schneider’s S2 cells was performed as previously described^5, 31^. For scans of images of full blots see the Supplementary Figs S1 and S2.

### Free CoA measurements

Tissue samples (~100 mg) were homogenized in 1 ml ice cold MilliQ water using 2.3 mm Zirconia/Silica beads and Precellys beadbeater system (6000 rpm; −2 × 15 sec). Samples were centrifuged at 16,000 × g at 4 °C for 20 min to collect the supernatant. To 10 µl tissue homogenate, 5 µl TCEP 50 mM was added and incubated for 15 minutes at room temperature. Subsequently, 8 µl ammonium hydroxide (12.5%) and 50 µl saturated ammonium sulfate were added to each sample and centrifuged for 5 min at 5500 rcf. To each supernatant, 300 µl MeOH/ACN (methanol/acetonitrile) was added. Appropriate dilution series of CoA standards were similarly processed before analysis. Extracts were injected onto Shimadzu Nexera HPLC system equipped with a Sciex API 5500 mass spectrometer from Applied Biosystems. The analytical column used was a SeQuant ZIC cHILIC guard column (20 × 2.1 mm) connected to a Merck seQuant ZIC cHILIC column (3 µm, 100 × 4.6 mm). The mobile phase consisted of solvent A: 5% acetonitrile in milli-Q water; solvent B: 200 mM ammonium acetate pH 4.5 and solvent C: acetonitrile. The following LC solvent program (1.2 mL/min; 40 °C) was used, where solvent B was kept at 20%: [0–1 min]: 5% solvent A, 75% solvent C; [1–3 min]: 20% solvent A, 60% Solvent C; [3–6 min]: 20% solvent A, 60% solvent C; [6–6.1 min]: 5% solvent A, 75% solvent C; [6.1–9 min]: 5% solvent A, 75% solvent C. The tandem mass spectrometry system was operated in positive ion mode. The detailed mass spectrometer conditions were as follows: probe temperature, 700 °C; ionization spray voltage, 5500 V; Turbo Ion Spray gas 1, 40 psi; collisionally activated dissociation gas, nitrogen, adjusted at 8 on a Sciex scale of 0–12; curtain gas, nitrogen, adjusted at 25 psig. The following multiple-reaction monitoring transitions were optimized: m/z 768 → 261 (CoA).

### Electron microscopy

Small pieces of liver tissues were fixed in 2% glutaraldehyde and 0.1 M sodium cacodylate (pH 7.4) and stored at 4 °C until usage. Pieces of 3mm^3^ were then generated and incubated in fresh 2% glutaraldehyde and 0.1 M sodium cacodylate (pH 7.4) for 24 hrs. Samples were rinsed twice in 0.1 M sodium cacodylate (pH 7.4) and subsequently post-fixed in 1% osmium tetroxide (OsO4) and 1.5% potassium ferrocyanide (K_4_Fe(CN)_6_) in 0.1 M sodium cacodylate at 4 °C for 2 hrs. Samples were rinsed in ddH_2_O, dehydrated in a graded ethanol series until 100% ethanol. Ethanol was replaced by acetone (RT) and kept in 1:1 ratio of epoxy resin: acetone while rotating (RT) for 10 hours. Epoxy resin (50%) was replaced with 100% resin for 30 min (RT) while rotating (3X). Samples were placed for 20 min at 58 °C and 1 hour at 37 °C under low pressure (200 mbar). Epoxy resin was polymerized at 58 °C. Ultrathin sections were placed on copper grids, and uranyl acetate in methanol followed by lead citrate were used to contrast the sections. Images were taken by transmission electron microscope (Philips CM100).

### Statistical analysis

Specific information per experiment is provided in the Figure Legends. All measurements were performed in triplicate at a minimum, and for all studies at least 3 mice were used per condition and measurement, unless indicated otherwise.

## Results

### Synthesis of P-PantSAc

**Figure 2:**
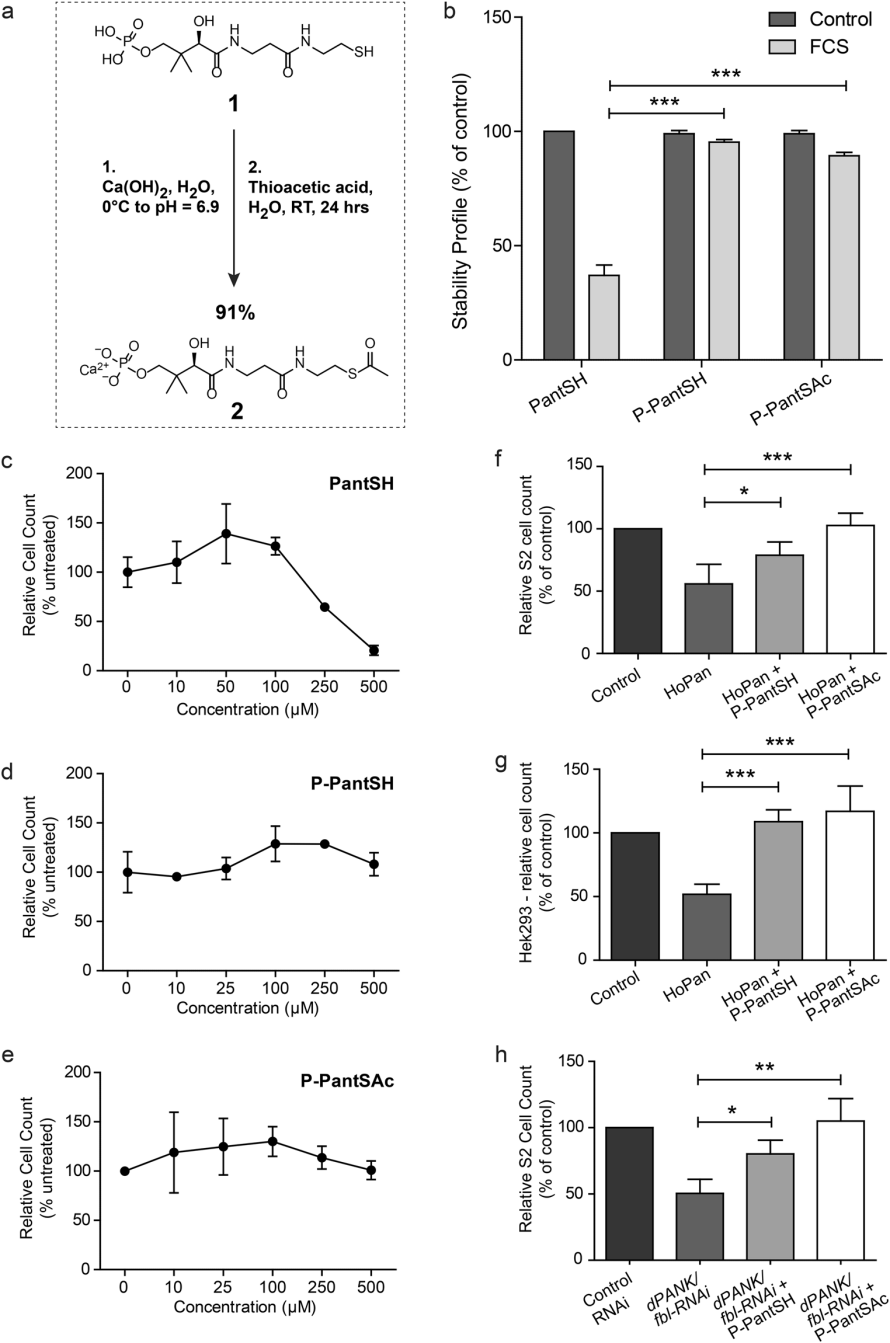
Synthesis, characteristics and rescue potential of P-PantSAc. (**a**) Schematic synthesis route of P-PantSAc. (**b**) Stability of P-PantSAc in foetal calf serum at 37 °C was determined using HPLC analysis and compared to the known compounds PantSH (non-serum-stable control) and P-PantSH (stable-serum control). Stability in serum for each compound is given as % of stability in PBS. For stability measurement in PBS n = 2 samples were measured. (**c**-**e**) Toxicity of PantSH, P-PantSH and P-PantsAc, respectively, was tested in HEK293 cells by determining cell numbers after 4 days of incubation with the compounds using increasing concentrations. (**f**-**h**) Rescue potential of P-PantSAc was tested in cells with impaired PANK activity using HoPan in S2 cells (**f**) or in HEK293 cells (**g**) or in cells treated with dPANK/fbl RNAi (**h**), as a control an RNAi construct was used specific for a irrelevant human gene hMAZ. (For all graphs mean ± SD is given. Two-tailed Student t-test was performed. *p < 0.05, **p < 0.01, ***p < 0.001).

In order to investigate the possibility of acetylation of the free thiol of P-PantSH (Fig. 2a), this molecule was synthesized as previously described^29^, with the intention of direct synthetic transformation to P-PantSAc (Fig. 2a). Initial acetylation attempts of P-PantSH with thioacetic acid resulted in <50% conversion, despite use of excess reagent or elevated temperatures (data not shown). As an alternative approach, in order to stabilize the free phosphate we converted the P-PantSH to the calcium salt which was subsequently treated with thioacetic acid (Fig. 2a). This resulted in complete conversion during acetylation as determined by LC-MS. Following purification, P-PantSAc was obtained as a calcium salt in excellent yields of (91%), with a purity of 95%, and retained favorable physical properties including high water solubility, which facilitated characterization of biological activity.

### Stability and toxicity of P-PantSAc

Since PantSH and specific PantSH derivatives are degraded in serum by the presence of pantetheinases^22–26^, we first investigated the stability of P-PantSAc in the presence of serum. This experiment was done in parallel with PantSH (which is not stable in serum) and P-PantSH (which is stable in serum)^29^ that served as positive and negative controls. Each compound was incubated for 30 min at 37 °C in Fetal Calf Serum (FCS). The HPLC analysis indicated that P-PantSH and P-PantSAc were stable in the presence of FCS, with 96% and 91% respectively of the compounds still present in the sample after incubation (Fig. 2b). These results showed that P-PantSAc has increased stability in serum compared to PantSH, of which levels were reduced to 36% after incubation in FCS. The addition of the acetyl group did not influence the serum-stable characteristics of P-PantSH as P-PantSAc showed comparable stability to the parent compound.

Next, we compared the toxicity of increasing concentrations of PantSH, P-PantSH and P-PantSAc in a cell-based assay. Human embryonic kidney cells (HEK293) were cultured under normal conditions allowing exponential cell growth, with and without addition of the compounds in the growth medium in various concentrations. After 4 days the cell count was determined. Whereas increasing concentrations (>100 µM) of PantSH clearly resulted in decreased cell proliferation, P-PantSH and P-PantSAc did not show toxicity up to 500 µM (Fig. 2c–e). The observed toxicity of PantSH is consistent with a previous study^18^.

### Rescue potential of P-PantSAc in cells

The acetylation of the terminal thiol of P-PantSH did not alter the stability or toxicity of the parent compound. We subsequently tested whether P-PantSAc possesses rescue abilities of conditions induced by impaired CoA biosynthesis comparable to that of P-PantSH^29^. Cells were treated with the PANK inhibitor HoPan, a Pan antimetabolite, which inhibits phosphorylation of Pan by PANK, leading to decreased biosynthesis and levels of CoA^29, 34^. Consistent with previous reports, HoPan addition was associated with decreased cell viability^29, 34^. P-PantSH and P-PantSAc both rescued the decreased cell count induced by addition of HoPan to the growth medium. This effect was observed in insect Schneiders’ S2 cells (Fig. 2f) and in the mammalian cell line HEK293 (Fig. 2g). In addition, downregulation of *Drosophila* PANK (dPANK/fbl) by RNAi also resulted in decreased cell count, and addition of P-PantSAc normalized cell counts back to control levels (Fig. 2h).

**Figure 3:**
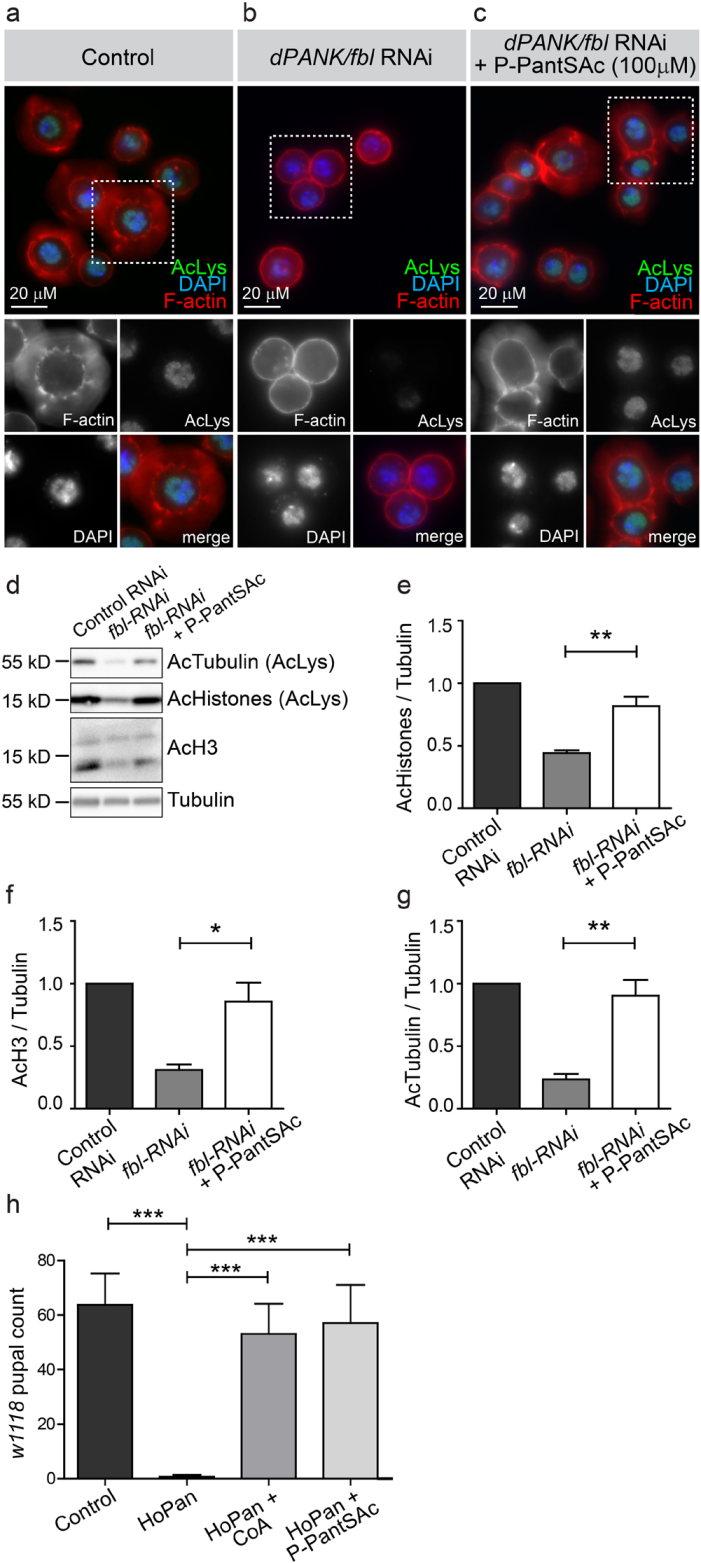
P-PantSAc rescues decreased acetylated lysine levels in dPANK/fbl RNAi treated cells and HoPan-induced larval lethality. (**a**) Control *Drosophila* S2 cells show normal levels of histone acetylation visualized by immunofluorescence using antibodies specific to acetylated lysines (AcLys in green). Rhodamin-phalloidin was used to visualize actin (F-actin in red), and DAPI was used to stain nuclei (in blue). (**b**) dPANK/fbl RNAi was used to downregulate levels of *Drosophila* PANK inducing a decrease of lysine acetylation levels. (**c**) Addition of P-PantSAc to the medium of dPANK/fbl RNAi-treated cells restored lysine acetylation levels. Bar represents 20 µm. (**d**) Western blot analysis for Acetylated Tubulin levels and Acetylated histone levels of control cells, dPANK/fbl RNAi treated cells and dPANK/fbl RNAi treated cells supplemented with P-PantSAc. An antibody against Acetylated lysines (AcLys) was used to detect Acetylated Tubulin and Acetylated histones. An antibody specific for Acetylated histone H3 was also used to detect levels of Acetylated Histone 3 (AcH3). Tubulin was used as a loading control. (**e**-**g**) Quantifications of (**d**). (For all graphs mean ± SEM is given. Two-tailed Student t-test was performed. *p < 0.05, **p < 0.01, ***p < 0.001). (**h**) HoPan provided via the food of developing *Drosophila* larvae induced lethality. Larval lethality was rescued by co-addition with HoPAN of CoA or P-PantSAc at the pupal stage. (For 3D mean ± SEM is given. Two-tailed Student t-test was performed. *p < 0.05, **p < 0.01, ***p < 0.001).

AcCoA/CoA levels determine lysine acetylation levels, and cells treated with HoPan show decreased levels of acetylated lysine^29, 31^. We tested whether addition of P-PantSAc also could rescue this cellular phenotype induced by impaired biosynthesis of CoA. Levels of acetylated lysine, as visualized by immunohistochemistry and Western blot analysis, were decreased under conditions of dPANK/fbl RNAi treatment (Fig. 3a–g) and were restored after addition of P-PantSAc (Fig. 3a–g), comparable to levels in the control (Fig. 3a–g).

To further investigate the potential of P-PantSAc, we investigated whether the compound could restore HoPan-induced lethality in an animal model of compromised CoA biosynthesis. *Drosophila* larvae were fed HoPan, which induced lethality early during development as previously reported^29^. For all cell-based assays done, synthetically prepared P-PantSH was used as a positive control (Fig. 2b–h). However, availability of P-PantSH is limited and its synthesis challenging. Therefore in all *in vivo* experiments (flies and mice) requiring relatively large amounts of P-PantSH, commercially purchased CoA was used as a source of P-PantSH in the positive control. In *Drosophila* and mice, CoA is converted to P-PantSH, and the rescue potential of P-PantSH similar to that of CoA^29^. Comparable to CoA, P-PantSAc fully rescued HoPan-induced larval lethality (Fig. 3h). This supports the finding that P-PantSAc is a stable non-toxic compound able to rescue various phenotypes induced by impaired PANK-dependent CoA *de novo* biosynthesis in insect and mammalian cells and in *Drosophila* larvae.

### Rescue potential of P-PantSAc in HoPan treated mice

**Figure 4:**
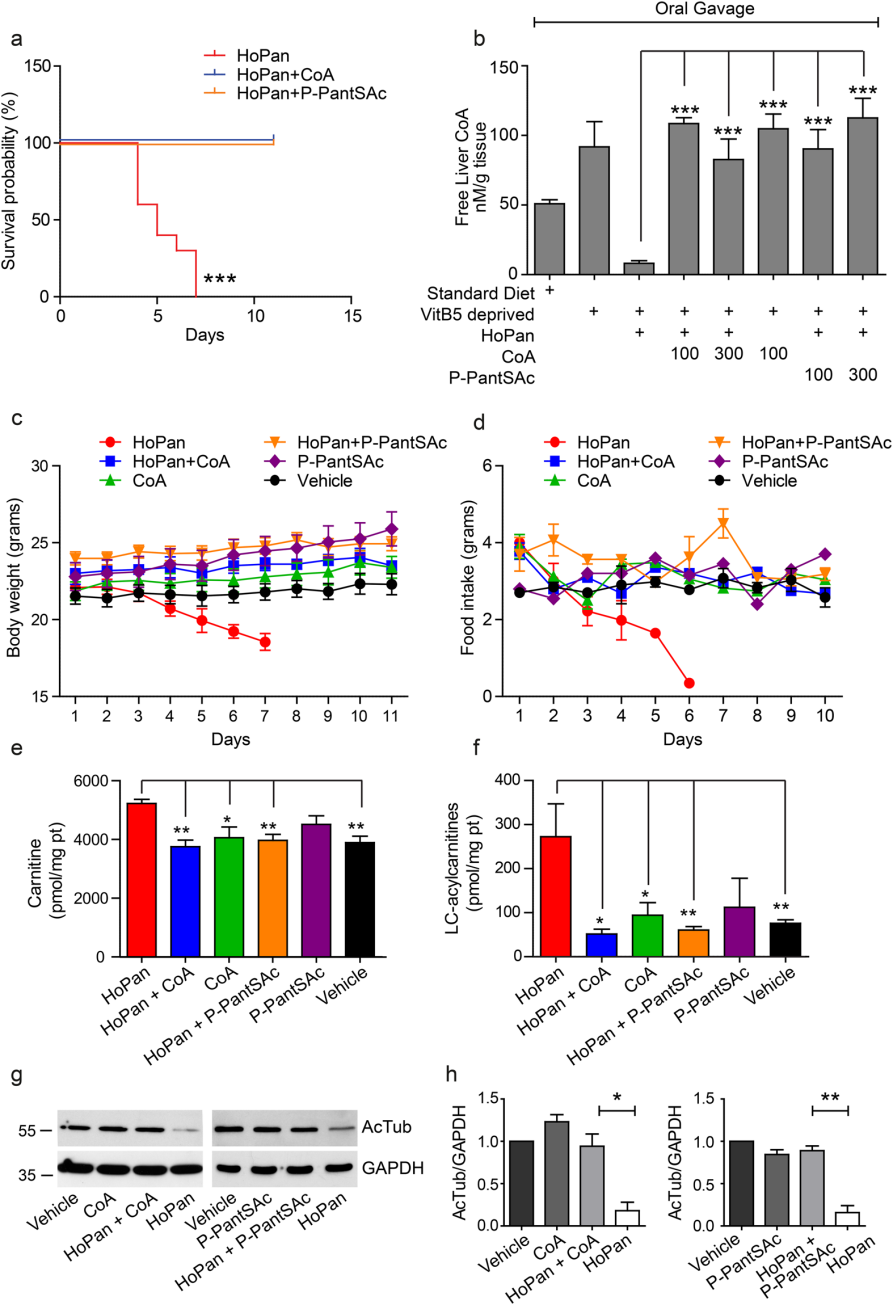
CoA and P-PantSAc rescue phenotypes induced by HoPan in mice. (**a**) Mice were treated with HoPan, HoPan + CoA, or HoPan + P-PantSAc, and survival was determined. Survival curves are shown as Kaplan-Meier plots. Mice treated with HoPan alone had a median survival of 5 days, whereas mice treated with HoPan + CoA, HoPan + P-*PantSAc* survived till the end of the experiment at Day 11. (**b**) Liver samples from treated and untreated mice were isolated, and CoA levels were measured using mass spectrometry. (For 4b mean ± SEM is given). CoA measurements in liver derived from mice treated with standard diet, Pan-deprived diet alone, Pan-deprived diet + HoPan, Pan-deprived diet + HoPan + 100 mM CoA, Pan-deprived diet + HoPan + 300 mM CoA, Pan-deprived diet + HoPan + 100 mM P-PantSAc, and Pan-deprived diet + HoPan + 300 mM P-PantSAc were determined. (**c**) Mice were treated with indicated compounds and weight was measured daily. Mouse weight is given in grams and represented as means ± SEM (for P-PantSAc treated mice, n = 2 mice were used). (**d**) Food intake was determined and given in grams of food intake per animal. Results are presented as mean ± SEM of at least 3 animals per treatment (for P-PantSAc, n = 2 animals were used). (**e**) Liver levels of free carnitine and (**f**) long-chain acylcarnitines, expressed in pmol/mg protein, are shown as mean ± SEM. (for P-PantSAc, n = 2 animals were used). (**g**) Western blot analysis of tubulin acetylation in livers from mice treated with HoPan, HoPan + CoA, CoA, Hopan + P-PantSAc, P-PantSAc, and vehicle is shown. GAPDH was used as loading control. A representative blot of three independent experiments is shown. (**h**) Quantification of g. For all panels mean ± SEM is given, statistically significant differences were determined by Student t-test. *p < 0.05, **p < 0.01, ***p < 0.001.

**Figure 5:**
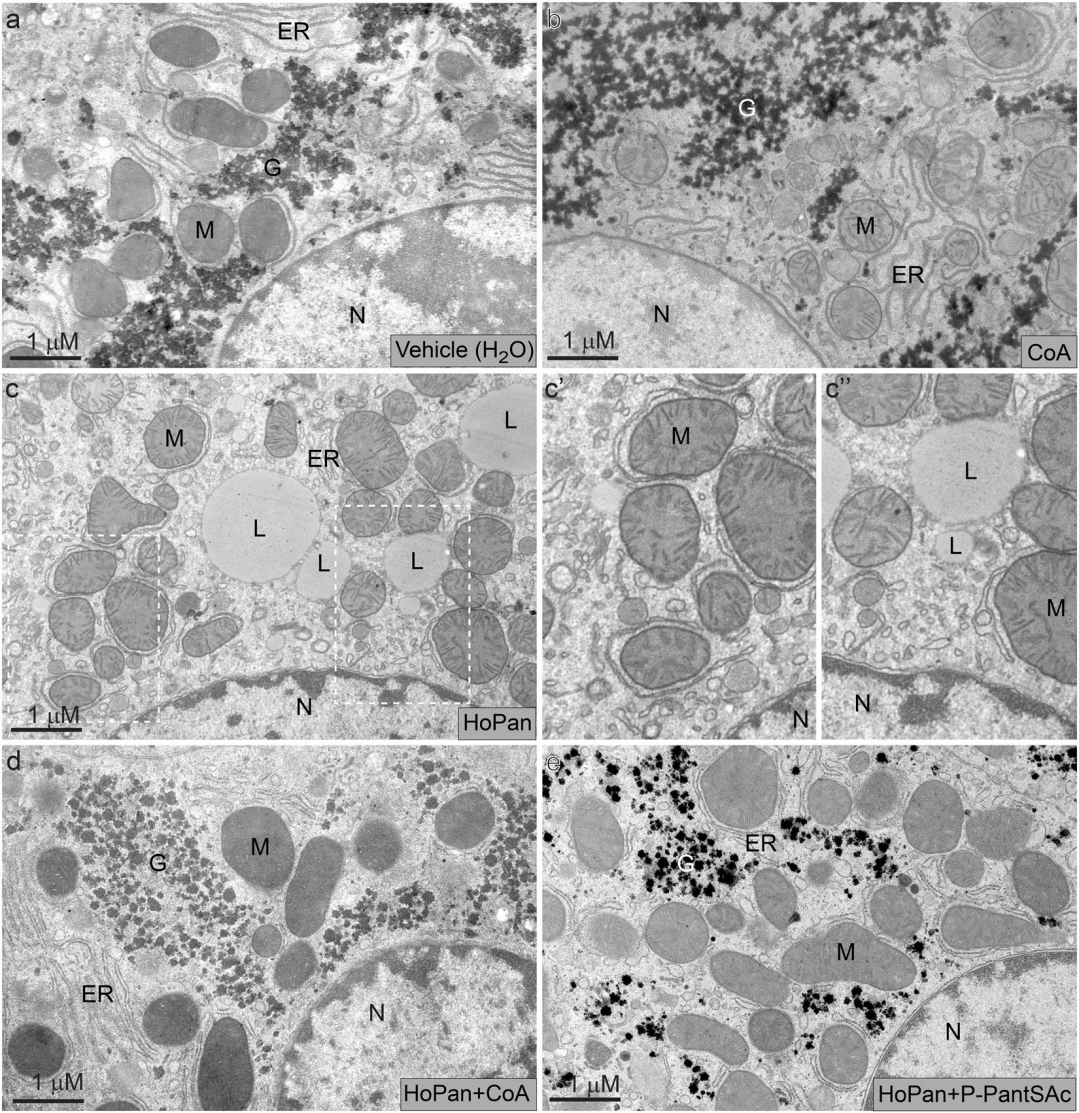
Ultrastructural changes in liver tissue induced by HoPan are prevented by P-PantSAc. Electron microscopy was used to analyze the ultrastructure of liver samples from untreated and treated mice. (**a**) Untreated mice. (**b**) Mice treated with CoA alone for 11 days. (**c**) Mice treated with HoPan for 5 days. (**c’**) Higher magnifications of inserts shown in (**c**,**d**) Mice treated with HoPan + CoA for 11 days. (**e**) Mice treated with HoPan + P-PantSAc for 11 days. ER = endoplasmic reticulum; G = glycogen; M = mitochondria; N = nucleus; l = lipid droplet. Scale bar represents 1 µm.

In order to test the rescue potential of P-PantSAc *in vivo* in an established mammalian setting, we used HoPan-treated mice as previously described^34^. For these experiments mice were kept on pantothenate deprived diet for 2 weeks before HoPan was provided^34^. HoPan-treated mice became lethargic after 5–6 days of treatment, requiring euthanasia (Fig. 4a). Measurement of CoA levels in liver tissue from 5 day-HoPan-treated animals revealed a large decrease in CoA levels compared to mice kept on normal diet or pantothenate deprived diet, consistent with previous reports^34^ (Fig. 4b). Remarkably, pantothenate deprived diet alone was associated with an increase in free liver CoA levels compared to control diet. An explanation for this effect is presently unavailable, however, it could be a compensatory mechanism present in the liver. Various concentrations of CoA and P-PantSAc were tested on HoPan-treated mice for their rescue potential. A dose of 100 μg/g/day of either CoA or P-PantSAc provided via oral gavage for 11 days fully restored liver CoA levels (Fig. 4b). This concentration was used for all subsequent experiments. In addition to their shortened lifespan and decreased liver CoA levels, HoPan-treated mice displayed reduced spontaneous movements (Supplementary Video S1), decreased body weight (Fig. 4c) and less food intake (Fig. 4d), increased levels of liver carnitine and acylcarnitines (Fig. 4e,f), and reduced tubulin acetylation (Fig. 4g,h **)**, all manifestations of severe CoA deficiency. During 11 days of simultaneous treatment with HoPan and either CoA or P-PantSAc, mice maintained their weight, remained healthy, and exhibited normal exploratory behavior (Fig. 4a–d; Supplementary Video S2). Moreover, levels of carnitine and long-chain acylcarnitines and the level of tubulin acetylation were similar to levels from control mice treated with vehicle alone (Fig. 4e–g,h). These results provide direct evidence that P-PantSAc is able to protect against HoPan-induced impairment (Fig. 4e–h, and Supplementary Video S3) and is comparable to the protection using CoA. The HoPan-induced phenotype in mice arises primarily from the effect of HoPan on liver^34^. Indeed electron microscopic analysis of liver tissue demonstrated that HoPan induces the formation of lipid droplets, depletes glycogen in liver, and dilates the endoplasmatic reticulum, all features that are prevented by oral feeding of CoA or P-PantSAc to the mice (Fig. 5). The previously reported HoPan-induced disorganization of mitochondrial cristae^34^ was not observed in our study. No toxicity from P-PantSAc or CoA was observed in the treated mice. Moreover when mice were treated with 100 µg/g of P-PantSAc intraperitoneally every other day for 75 days, no differences were observed in weight gain or in the histology of liver, heart, brain and testis compared to vehicle-treated mice (data not shown). These results demonstrate that P-PantSAc is able to prevent CoA depletion and its associated phenotypes in HoPan treated mice.

## Discussion

We describe the synthesis, purification and characterization of the P-PantSH derivative, P-PantSAc, a serum-stable compound that replenishes CoA/AcCoA levels and prevents phenotypes associated with PANK deficiencies. The rescuing characteristics of P-PantSAc are comparable to those described for P-PantSH and CoA^20, 29, 35^(and this manuscript), suggesting that all three molecules have therapeutic potential for PKAN. P-PantSAc may have advantages because the synthesis procedure allows for linkage of other acyl moieties to P-PantSH in order to convert P-PantSH into various acyl derivatives. P-PantSAc can be converted into AcCoA by the CoA machinery from bacteria^30^, suggesting that levels of any acyl-CoA of choice could be increased by adding specific P-PantS-Acyl forms to cells or organisms. This enables influencing numerous metabolic processes that require specific acyl-CoAs; however, further research is needed to understand the potential impact of P-PantSAc and other P-PantSAcyl forms on AcCoA/CoA ratios and levels, intermediary metabolism and other critical cellular processes.

In this manuscript we focus on the potential of P-PantSAc to prevent phenotypes induced by impaired function of the first step (*i.e*., Pan phosphorylation by PANK) in the canonical CoA *de novo* biosynthesis pathway. One effective strategy to block this step is treatment of cells or mice with HoPan^34^. In mice, HoPan inhibits all PANK activity and induces a more dramatic liver-centric phenotype compared to impairment of single PANK isoforms via genetic manipulations. This explains why HoPan treated mice survive for only 5–6 days whereas *Pank2* ^*−/−*^ or *Pank1* ^*−/−*^ single homozygous knockout mice are viable^36, 37^. Therefore, HoPan treated mice may be considered to be an exaggerated biochemical model of PKAN, and any compound that is able to prevent its severe phenotype has potential to also prevent the impaired function associated with defective PANK2, as in PKAN. However, the addition of Pan has been shown to rescue HoPan induced phenotypes^34^, therefore our results do not exclude the possibility that in mice *in vivo* P-PantSAc is degraded leading to Pan mediated rescue. Nevertheless, our results demonstrating that P-PantSAc also prevents various phenotypes induced by eliminating PANK using RNAi, support the rescue potential of P-PantSAc directly. Moreover, the possibility of direct conversion of P-PantSAc to AcCoA via COASY^30^, suggests a strong translational potential of P-PantSAc towards clinical applications. Next, it will be important to investigate whether P-PantSAc can rescue other genetic models for PKAN such as: the fruitfly model^8, 18, 29, 31^, a zebrafish model^20^ and iPS-derived neurons generated from PKAN patients^35^, models in which CoA was proven to be successful.

Because PKAN is a disorder of the central nervous system, another critical follow-up study is to investigate whether P-PantSAc is able to cross the blood-brain barrier. Limited information is available concerning blood-brain barrier-passing capacities of CoA precursors. Specific synthetic modifications of P-Pan, phosphopantothenate aryl phosphoramidate derivatives, provided to *Pan1* ^*−/−*^ mice via the oral, intraperitoneal or intravenous routes resulted in restoration of decreased levels of CoA in liver but not in brain^38^. P-PantSH has the ability to cross biological membranes *in vivo* and artificial membranes *in vitro* that mimic the blood-brain barrier^29^. It remains to be investigated whether cellular uptake of P-PantSAc is comparable to P-PantSH.

In conclusion we demonstrate the successful synthesis of the pantetheine-derivative P-PantSAc, its serum-stability and low toxicity, and its rescue potential in various *PANK* deficient models. Finally, the availability of P-PantSAc and other P-PantS-Acyl forms will be valuable tools to interrogate the cellular physiology of CoA and CoA derivatives.

## Electronic supplementary material

Supplementary Figures S1 and S2

Supplementary Video 1

Supplementary Video 2

Supplementary Video 3
