# Supplementary material for: Acetyl-4′-phosphopantetheine is stable in serum and prevents phenotypes induced by pantothenate kinase deficiency
Source: Sci Rep. 2017 Sep 12;7:11260. doi: 10.1038/s41598-017-11564-8 (PMC5595861; doi:10.1038/s41598-017-11564-8)
Supplement: Supplementary file 1 — Supplementary Figures S1 and S2 [file 41598_2017_11564_MOESM1_ESM.pdf]

Supplementary Information

Acetyl-4'-phosphopantetheine is stable in serum and prevents phenotypes induced by pantothenate kinase deficiency.

Ivano Di Meo, Cristina Colombelli, Balaji Srinivasan, Marianne de Villiers, Jeffrey Hamada, Suh Y. Jeong, Rachel Fox, Randall L. Woltjer, Pieter G. Tepper, Liza L. Lahaye, Emanuela Rizzetto, Clara H. Harrs, Theo de Boer, Marianne van der Zwaag, Branko Jenko, Alen Čusak, Jerca Pahor, Gregor Kosec, Nicola A. Grzeschik, Susan J. Hayflick, Valeria Tiranti, Ody C.M. Sibon

Supplementary Figure 01

Original blots used for Figure 3d-g

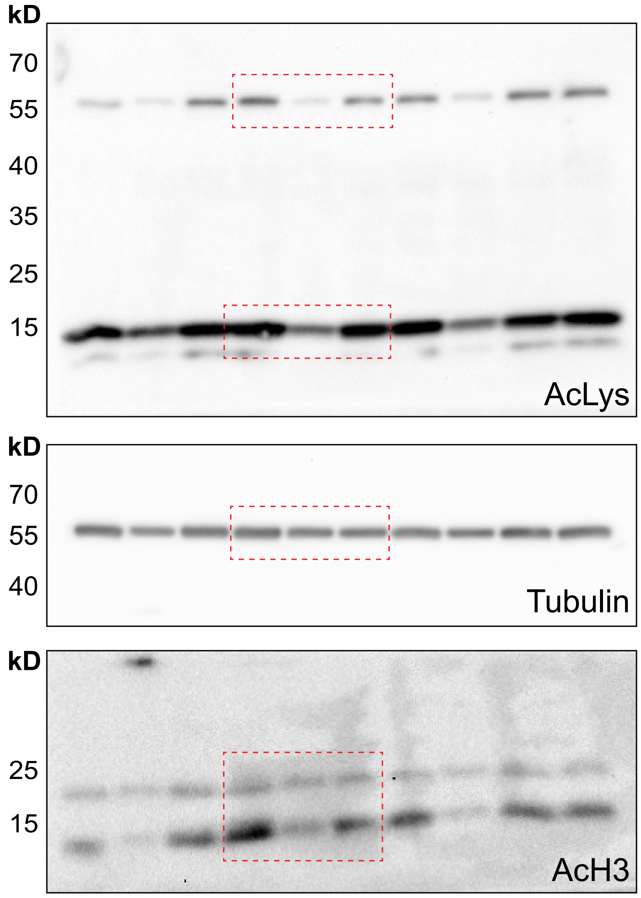

Supplementary Figure 02

Original blots used for Figure 4g

a

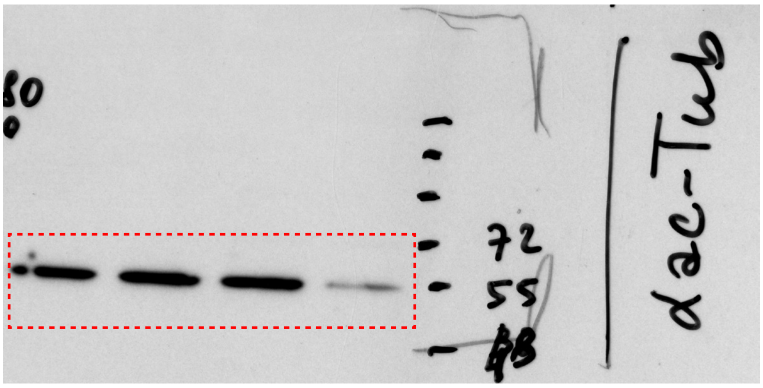

anti-AcTubulin

b

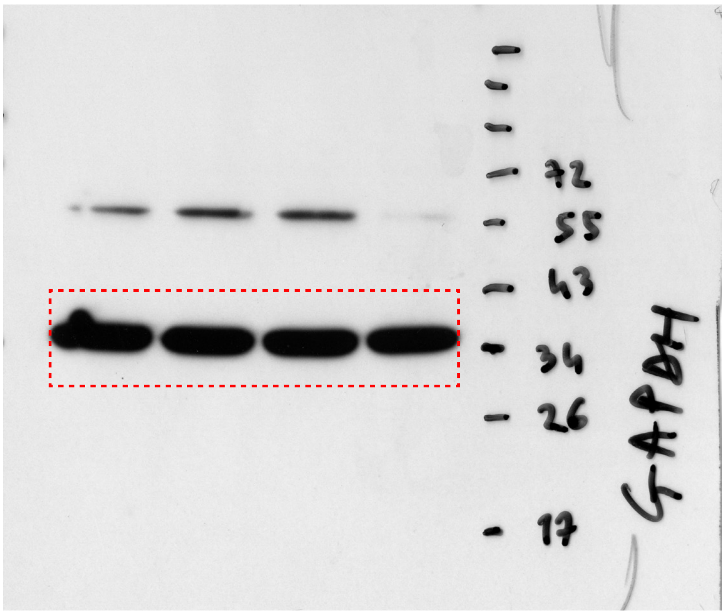

anti-GAPDH

c

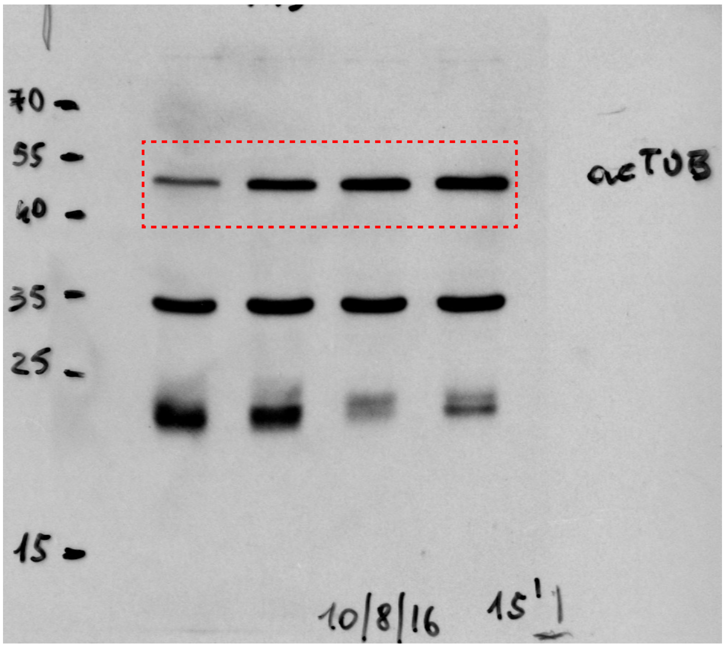

anti-AcTubulin

d

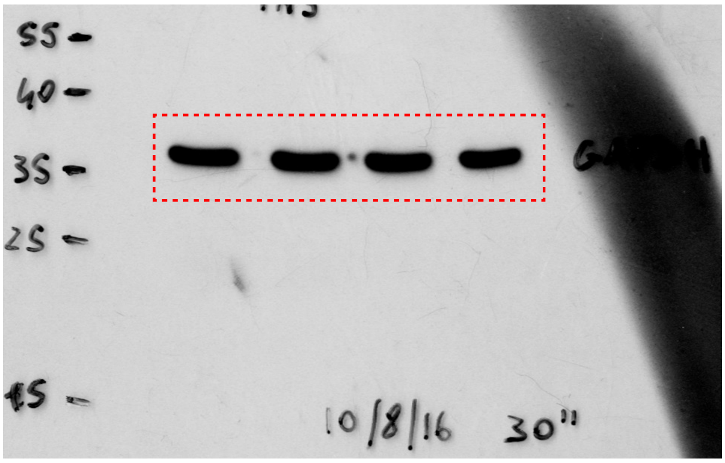

anti-GAPDH
